# Supplementary material for: Shifting parental beliefs about child development to foster parental investments and improve school readiness outcomes
Source: Nat Commun. 2021 Oct 1;12:5765. doi: 10.1038/s41467-021-25964-y (PMC8486787; doi:10.1038/s41467-021-25964-y)
Supplement: Supplementary file 1 — Supplementary information [file 41467_2021_25964_MOESM1_ESM.pdf]

Shifting parental beliefs about child development to foster parental investments and improve school readiness outcomes

Supplementary Information

John A. List Julie Pernaudet Dana L. Suskind

1 Economic framework

The goal of our set of experiments is to assess the role played by parental beliefs in the development of children's skills. In this section, we present the hypotheses we test within the production technology framework developed by Cunha et al.[1]. In their model, the law of motion for skill formation follows some function that depends on the stock of skills and parental investments at the previous period, θ_t and I_t (with t referring to children's age - in our case $t \in \{6, 9, 12, 18\}$ months for the newborn experiment and $t \in \{30-36, 36-42\}$ for the home-visiting experiment), and on parental characteristics (e.g., education, IQ, race, etc.), that we denote $\mathbf{X}$:

$$\theta_{t+1} = \mathrm{f_t}(\theta_t, I_t, \mathbf{X})$$

In our framework, instead of taking parental investments in children as given, we go one step further in the understanding of the primitives of early human capital formation and model parents' investments as a function of their contemporaneous and past beliefs about child development, ϕ_t and $\phi_{t-\ell}$, as well as their characteristics and child's skills:

$$I_t = \mathrm{g_t}(\phi_t, \phi_{t-\ell}, \theta_t, \mathbf{X})$$

The experiments we designed generate an information shock and are therefore expected to generate exogenous variation in parental beliefs, allowing us to test the sign of the partial derivatives:

$$\frac{\partial I_t}{\partial \phi_t} \gtreqless 0 \qquad \text{and} \qquad \frac{\partial I_t}{\partial \phi_{t-\ell}} \gtreqless 0$$

The closest paper including parents' beliefs in the production function of children's skills is a paper by Boneva and Rauh[2]. Combining survey data eliciting beliefs about the returns to parental investments with the British Cohort Study data, the authors estimate a structural model of skill

production and simulate the effects of a shift in parental beliefs on investments and long-run economic outcomes of the child. In this paper, we take a reduced-form approach that exploits the randomly assigned treatment as an instrument for beliefs in a Two-Stage Least Squares (2SLS) strategy. After checking that the treatment indeed induces a shift in beliefs in the first stage (checking that the instrument is not weak), we regress parents' investments on the predicted beliefs. The empirical implementation and results are presented in section 2.

2 Do changes in parental beliefs about child development cause changes in parental investments in children?

This section explores further the relationship between the changes in parental beliefs about early skill formation and the changes in parental investments in children (see section 1 for a full presentation of our economic framework). To do so, we exploit the random variation induced in parents' beliefs by each intervention in an attempt to estimate the causal effect on parents' investments via 2SLS regressions.

In the first stage, we regress our measure of beliefs on the treatment status variable. In the second stage, we regress our measures of parental investments on the predicted beliefs recovered from the first stage. This technique yields an estimate of the effect of parents' beliefs on their investments that can be interpreted causally if the treatment status strongly predicts beliefs (which is verified in Table 1), and if the treatment has no direct impact on parents' investments (no impact other than through beliefs improvement). Although the second condition cannot be directly tested, we argue that since the interventions mainly consist of providing information about child development and parents' potential to affect it, and of teaching parents how to improve their parenting practices, it is plausible that one of the main channels through which the interventions affect parents' investments in their child is by changing their beliefs and knowledge of parenting. This assumption is stronger in the case of the home visiting program and we discuss other potential channels at the end of this section.

Supplementary Table 1 presents the results at the same time points as Table 1. The first four columns of the table show the impact of beliefs measured at 6, 7, 12, and 18 months on parental inputs measured at respectively 6, 9, 12, and 18 months, using the Newborn data. The last two columns show those impacts at different ages, using the home-visiting data. At t=6 months, we fail to reject the hypothesis that changes in parents' beliefs lead to changes in their investments (hypothesis $\frac{\partial I_t}{\partial \phi_t} = 0$ in our economic framework, Section 1 of Supplementary Information), which is consistent with the fact that we find no impact of the intervention on investments at that age (Table 1). However, the second row of the table reveals that improved knowledge of the impact of parental investments at 6 months leads to improved parental investments at 9 months: $\frac{\partial I_t}{\partial \phi_{t-\ell}} > 0$, for t=9 months, ℓ= 3 months (if we decompose the measure of parental investments, we find that

the overall impact is driven by improvement in parents' sensitivity to cues, social-emotional and cognitive growth fostering - results available upon request). Similarly, the home-visiting data reveal that improvements in parents' beliefs at 30-36 months induce contemporaneous improvements in their investments, consistent with the treatment effect on parents' investments we find at that age in Table 1. Other impacts shown in the table all have a positive sign, but are too small to be significant given the number of observations we have. These results support the hypothesis that the impacts we find on parents' investments at 9 months in the Newborn experiment and at 30-36 months in the home-visiting experiment are driven by learning.

To take into account the possibility that the interventions also affect parental investments in children through other channels such as social pressure (by communicating norms about what "good parents" should do) or accountability (due to the monitoring of their behavior by the research team), we re-did the estimations controlling for parents' perceptions of the pressure they face (measured by the TOol to measure Parenting Self-Efficacy (TOPSE) - Pressure subscale). Results remain similar in the Newborn program but shrink and become statistically non-significant in the HV program, suggesting that the exclusion condition described above might be less plausible in the latter case (table available upon request).

Supplementary Table 1: Impact of parents' beliefs about the impact of parental investments on their investments at different time points

	Newborn program				HV program	
Beliefs at:	6m	7m	12m	18m	30-36m	36-42m
Investments at:						
6m	0.16					
	0.21					
	363					
9m	0.35**	0.31				
	0.03	*0.20*				
	340	257				
12m	0.16	0.22	0.17			
	0.29	*0.36*	*0.38*			
	362	272	372			
18m	0.12	0.14	0.15	0.28		
	0.43	*0.61*	*0.53*	*0.36*		
	312	232	308	321		
30-36m					0.45*	
					0.06	
					61	
36-42m					0.33	0.37
					0.17	*0.18*
					59	60

Note: Each row gives the change in parents' investments (in standard deviations of the control group) that is associated with an increase of the beliefs score by one standard deviation (the beliefs score is instrumented with the treatment status variable). The second row gives the corresponding p-value in italics based on a two-sided Student's t test without adjustment for multiple comparisons. * for p-value<0.1, ** for p-value<0.05, *** for p-value<0.01. The third row gives the number of observations.

3 Balancing checks

Supplementary Table 2: Balancing checks at baseline

	Newborn program			HV program		
Variable	Mean C	T-C	N	Mean C	T-C	N
1 if child is a girl	0.53	0.00	475	0.44	-0.12	91
		0.96			*0.25*	
Parent's age	26.01	1.01*	475	33.00	0.48	91
		0.06			*0.77*	
Highest educ. level < High-school dip.	0.32	-0.05	475	0.33	0.06	91
		0.24			*0.57*	
Number of children	2.17	0.09	469	2.67	0.05	91
		0.42			*0.85*	
Household income per month < $2 655	0.83	0.01	475	0.80	0.03	91
		0.73			*0.75*	
Primary language is English	0.65	-0.01	469	.	.	.
		0.90			*.*	
Black or African American	0.42	0.03	469	.	.	.
		0.55			*.*	
White	0.26	-0.01	469	0.29	0.10	91
		0.77			*0.31*	
Hispanic or Latino	0.54	-0.01	469	1.00	.	91
		0.89			*.*	
Medicaid/Medical card	0.85	0.03	469	0.96	-0.07	90
		0.35			*0.24*	
LINK card	0.52	0.07	469	0.62	-0.06	91
		0.15			*0.59*	
WIC supplements	0.70	-0.04	469	0.64	-0.12	91
		0.41			*0.24*	
Currently employed	0.35	-0.07*	469	0.51	-0.32***	91
		0.10			*0.00*	
Full-time student	0.05	-0.03	469	0.04	-0.04	91
		0.15			*0.16*	
Most recent job full-time	0.33	0.06	468	0.53	0.02	90
		0.18			*0.84*	
Most recent job part-time	0.37	-0.01	468	0.31	-0.07	90
		0.85			*0.49*	
Recent traumatic event	0.24	0.08*	475	0.44	-0.10	91
		0.06			*0.35*	
Beliefs about impact of parental inputs	43.99	0.69	471	46.50	1.57	82
		0.41			*0.43*	

Note: Mean C gives the mean of the variable in the control group, T-C gives the difference with the treatment group. Below in italics is the corresponding p-value based on a two-sided Student's t test without adjustment for multiple comparisons. N gives the number of observations. * for p-value<0.1, ** for p-value<0.05, *** for p-value<0.01.

4 Measurement tools

This section presents the different measurement tools used for the analyses. Supplementary Table 3 below first provides a summary of all the variables used in the paper, explaining where they were used in the analysis and how they were measured. We then present in detail the content and format of each measurement tool.

Supplementary Table 3: Summary of the measures used in the analysis

Variable	Study	Used in	Measurement
**Parents' beliefs:**			
SPEAK	- Suskind et al. (2018)	Figure 1	Self-report
	- Newborn program	Table 1, Suppl. Tables 1, 4, 6, 7, Suppl. Fig. 1	
	- HV program	Table 1, Suppl. Tables 1, 5, 6, Suppl. Fig. 1	
	- Longitudinal	Table 2	
TOPSE	- Newborn program	Suppl. Tables 4, 7	Self-report
	- HV program	Suppl. Table 5	
TOI	- Newborn program	Suppl. Tables 4, 7	Self-report
	- HV program	Suppl. Table 5	
**Parents' inputs:**			
NCAST	Newborn program	Table 1, Suppl. Tables 1, 4, 6, 7	Direct obs.
LENA	HV program	Table 1, Suppl. Tables 1, 6	Direct obs.
**Child outcomes:**			
CDI I and II	Newborn program	Table 1, Suppl. Tables 6, 7	Self-report
ASQ-SE	- HV program	Tabls 1, Suppl. Table 6	Self-report
	- Longitudinal	Table 2	
PLS	Longitudinal	Table 2	Direct obs.
Woodcock-Muñoz	HV program	Table 1, Suppl. Table 6	
ROWPVT	HV program	Table 1, Suppl. Table 6	Direct obs.
PPVT	Longitudinal	Table 2	Direct obs.
Give N	HV program	Table 1, Suppl. Table 6	Direct obs.

Note: All variables except Woodcock-Muñoz and ROWPVT were analyzed as overall standardized scores. Standardization was made with respect to the mean and standard deviation of the full baseline sample distribution when the measure was available at baseline, and otherwise with respect to the distribution of the control group only at the earliest assessment point. For Woodcodk-Muñoz and ROWPVT, we built an index combining the two measures (average of the two z-scores).

Note that the full list of primary outcomes that were collected as part of the two experiments can be found on clinicaltrials.gov (references NCT02812017 and NCT03076268). In the Newborn study, we included in the paper all the primary outcomes observed repeatedly across the different assessment points as we are interested in assessing the evolution of the impacts. We thus excluded the StimQ Cognitive Home Environment and Child Behavioral Checklist, measured only once at later assessment points. In the home-visiting study, we included all primary outcomes.

4.1 Parental beliefs

SPEAK survey

The Survey of Parents' Expectations And Knowledge (SPEAK) is the tool we use to elicit parental beliefs about how parents' input impact child development for both the descriptive and impact analyses. Note that throughout the paper, we consider that parental beliefs are knowledge-based[3] and use the terms 'beliefs' and 'knowledge' interchangeably. The SPEAK survey has been developed by Suskind et al.[4] and focuses on beliefs about social-emotional and cognitive (with an emphasis on language) development of children between 0 and 5 years old. It consists of a series of questions that cover topics such as the age when babies can start being exposed to different types of reasoning (e.g., counting, sizes, shapes), the effect of media use or bilingualism on learning, the role of nature versus nurture in child early development, and best communication and reading practices. Parents have to answer on a four-point scale from "Definitely True" to "Definitely Not True" (except for questions about stages of development for which they had to select a stage, from 0 to 6 years old and more). The total score is simply the sum of the points over the series of questions. A higher score indicates a better understanding of the impact of parental inputs on child development. Parents complete the survey in English or Spanish, depending on their preferred language. Note that the survey is still in development and the formulation of some of the questions varies slightly between the version used to produce Figure 1 and the version used in the two experiments.
Few measurement tools are available in the literature to assess parental beliefs about child development at early stages. Cunha et al.[5], Attanasio et al.[6] and a series of related papers use hypothetical scenarios to elicit parental beliefs about the parametrization of the technology of skill formation. Here, we aim at assessing parental knowledge of early development more generally, covering a broad range of parenting practices that are relevant for the development of children's skills (media use, reading, responsiveness, languages spoken at home, etc.)

TOPSE survey

The second measure of parental beliefs we use is a scale called TOol to measure Parenting Self-Efficacy (TOPSE) that captures parental self-efficacy[7]. We use the short version of it[8]. It assesses parents' perception of their own ability to parent effectively in six dimensions: Emotion and Affection (e.g., "I can recognize when my child is happy or sad"), Play and Enjoyment (e.g., "I can plan activities that my child will enjoy"), Empathy and Understanding (e.g., "I am able to put myself in my child's shoes"), Pressure (e.g., "It is difficult to cope with other people's expectations of me as a parent"), Self-Acceptance (e.g., "I can be strong for my child"), and Learning and Knowledge (e.g., "I am able to learn and use new ways of dealing with my child"). Although each subscale can be studied independently, we only look at the overall score in the analysis, to limit the number of hypotheses we test. Note that the tool contains two other subscales, Control, and Discipline and Boundaries, that were not included in our survey as they were less directly relevant to the intervention and the duration of the assessment was constrained. Parents had to answer on a ten-point scale from "Completely Agree" to "Completely Disagree". Each item is scored between 0 and 10 points and the total score is the sum of all items. A higher score indicates higher parenting self-efficacy.

TOI survey

The Theory Of Intelligence (TOI) survey captures parents' beliefs about intelligence malleability of young children in a series of eight items with which parents have to say to what extent they agree, on a five-point Likert scale[9]. It focuses solely on the extent to which children can change their intelligence or ability, not on the elasticity of skills with respect to parental inputs (e.g., "A child's intelligence is something about them that they can't change very much").

4.2 Parent-child interactions

NCAST

To measure parents' inputs in the Newborn study, we rely on a direct assessment of parent-child interaction during a teaching episode, a measurement tool called the Nursing Child Assessment Satellite Training (NCAST) scale[10] that can be used for children as young as six months old. Each parent is filmed in a private room with his or her baby for 6 minutes while teaching a given task (e.g., stacking blocks, drawing). Then the video is coded by a trained external assessor along six subscales describing the quality of the interaction. The first four subscales describe the parent behavior towards the child: his or her sensitivity to cues (e.g., "Caregiver praises child's successes or partial successes"), response to child's distress (e.g., "Caregiver makes a positive, sympathetic, or soothing verbalization"), social-emotional growth fostering (e.g., "Caregivers makes cheerleading type statements to the child during the teaching interaction"), and cognitive growth fostering (e.g., "Caregiver responds to the child's vocalizations with a verbal response"). We sum those

four subscales in one single parental score of parent's interactions with the child for the main analysis (disaggregated results are presented in Supplementary Table 4). The last two subscales describe the child behavior towards the caregiver: clarity of cues (e.g., "Child makes clearly recognizable arm movement during the teaching episode (clapping, reaching, waving, pointing, ...)") and responsiveness to the caregiver (e.g., "Child vocalizes or babbles within five seconds after caregiver 's verbalization"). Similarly, we sum those two subscales in one single child score of child's interactions with the parent for the main analysis (disaggregated results are presented in Supplementary Table 4). For each of the 73 items, the assessor simply chooses between Yes and No, and the score is the sum of Yes answers. Those direct assessments of parent-child interaction give us detailed and objective measures of parental inputs that are relevant for children as young as 6 months old, and that have been found to predict later cognitive and psycho-motor outcomes [11,12].

LENA

In the home visiting program, parent-child interactions were measured using the Language ENvironment Analysis (LENA) technology. The measurement consists of using an audio-recording device that the child wears in a specially-designed t-shirt for approximately 8 hours on each recording day. The device records all sounds produced around the child and the data are then processed by a speech recognition software to generate three count-based metrics (see `www.lena.org`). In Table 1, parents' interactions with the child are measured through conversational turn counts (CTC) and child's interactions with the parents are measured through vocalization counts (CVC). We do not use the third metric called the adult word count (AWC) as this metric is not child-directed and counts any adult words around the child, including adults talking on the phone or talking to each other in the room, and does not correspond to an outcome of interest in our study, which focuses on parent-child interactions.

4.3 Children's skills

CDI

In the Newborn experiment, we measure child's vocabulary at 9, 12 and 18 months using the short form of the MacArthur-Bates Communicative Development Inventories [13] (CDI). The forms are filled out by parents and vary with age. At 9 and 12 months (level I), parents are presented with a list of words and have to indicate, for each word, whether their child understands it (receptive vocabulary) or understands and says it (expressive vocabulary). Both scores have been transformed in z-scores and averaged (disaggregated results are presented in Supplementary Table 4). At 18 months (level II), parents are presented with a list of words and have to indicate, for each word, whether their child says it (expressive vocabulary) and they answer a question asking whether their child is capable of combining words. Both questions have been transformed

in z-scores and averaged (disaggregated results are presented in Supplementary Table 4). Parents had to fill out the English form, and could additionally fill out the Spanish form, if their child was exposed to the two languages.

ASQ-SE

For the home-visiting experiment and Longitudinal study, we use the Ages and Stages Questionnaire: Social Emotional (ASQ-SE)[14] to measure social-emotional skills. The questionnaire is age-specific and completed by parents. They answer a series of questions such as "Does your child like to be hugged or cuddled?", "Does your child hurt himself on purpose?" on a three-point scale from "Never" to "Most of the time", with the possibility to indicate whether the described behavior is a concern for them. It is a tool used by pediatricians to detect potential issues in the social-emotional development of the child, when the overall score falls below a given threshold.

PLS

In the Longitudinal study (Table 2), the Preschool Language Scale (PLS) Fifth Edition was used to assess the language development of the children. The assessment is play-based, it is done by specifically trained assessors and covers a large range of linguistic skills that are specific to the age of the child[15].

Woodcock-Muñoz, ROWPVT and PPVT

In the home-visiting program, the Verbal Analogies test and the Picture Vocabulary test of the Woodcock-Muñoz Language survey were used to measure the expressive vocabulary of children[16]. The analogy test consists of a direct assessment in which a specifically trained assessor asks the child to complete a series of sentences (e.g., "A hairbrush is for hair and a toothbrush is for ..."). For the picture vocabulary test, the trained assessor points to a picture and asks the child to name the object (e.,g., a ball, a house, an apple).
To measure receptive vocabulary, the Receptive One-Word Picture Vocabulary Test (ROWPVT) Fourth Edition was used[17]. It is a direct assessment done by a specifically trained assessor who asks the child to point to specific objects or actions on an image that (s)he shows to the child. All scores were transformed in z-scores and aggregated in an overall vocabulary index (disaggregated results are presented in Supplementary Table 5).
The Longitudinal study (Table 2) uses another version of the picture vocabulary test called the Peabody Picture Vocabulary Test (PPVT)[18]. It is also a direct assessment of receptive vocabulary based on a series of images presented to the child.

Give-N task

To measure math skills, we use the Give N task, which consists of providing the child with a large bowl of small items and asking him or her to give a certain number of items (e.g., "Can you give me TWO bananas?").

5 Econometric model

For all our outcomes of interest, we estimate the following intent-to-treat model of being encouraged to watch the videos in the newborn study or receive home visits in the second study, separately for each assessment a:

$$Y_i^a = \alpha^a + \beta^a Z_i + \epsilon_i^a \quad (1)$$

where Z_i is a dummy variable indicating whether parent i was assigned to the treatment group rather than the control group, $a \in \{6, 7, 9, 12 \text{ or } 18 \text{ months}\}$ for the newborn study and $a \in \{30\text{-}36 \text{ or } 36\text{-}42 \text{ months}\}$ for the home-visiting study. The outcomes Y are all standardized with respect to the earliest time point they were measured. We use the full sample size when the earliest time point is pre-intervention and the control group sample only when the earliest time point is post-intervention. β should thus be interpreted as an effect size. We estimate the model separately for each assessment to allow the coefficients and unobserved shock to vary at each stage of development. In section 7, we present two variations of the specification shown in Supplementary Equation 1: one with control variables that have been selected based on the method described in Belloni et al.[19], and one that distinguishes control group parents who just received usual care from control group parents who were in the placebo group and watched a safety video for the newborn study. The results tables present p-values that are adjusted for multiple hypothesis tested. Those adjusted p-values are computed following the method described in List et al.[20]. The method consists of controlling for the risk of false positive at the level of a family of outcomes. We define one family as one type of outcome (belief, interaction or child outcome) at a given age. Following that rule, we have the following eleven families for the newborn study:

1. Beliefs about impact of parents' inputs at 6 months
2. Beliefs about impact of parents' inputs at 7 months
3. Beliefs about impact of parents' inputs at 12 months
4. Beliefs about impact of parents' inputs at 18 months
5. Interactions with child at 6 months + Interactions with parent at 6 months
6. Interactions with child at 9 months + Interactions with parent at 9 months

7. Interactions with child at 12 months + Interactions with parent at 12 months
8. Interactions with child at 18 months + Interactions with parent at 18 months
9. Vocabulary at 9 months
10. Vocabulary at 12 months
11. Vocabulary at 18 months

And the following six families for the home-visiting study:

1. Beliefs about impact of parents' inputs at 30-36 months
2. Beliefs about impact of parents' inputs at 36-42 months
3. Interactions with child at 30-36 months + Interactions with parent at 30-36 months
4. Interactions with child at 36-42 months + Interactions with parent at 36-42 months
5. Social-emotional skills at 30-36 months
6. Social-emotional skills + Vocabulary + Math skills at 36-42 months

Note that most our input and outcome measures correspond to indexes that aggregate several measurements (see Section 4 of Supplementary Information), which restricts (ex-ante) the number of hypotheses we test, hence the small size of our families and the limited (ex-post) adjustment we impose to the p-values. Non-aggregated results can be found in Section 6 of the Supplementary Information.

6 Additional impacts

Supplementary Table 4: Impacts of the Newborn program at different time points

	6 months		7/9 months		12 months		18 months	
	$\overline{T}-\overline{C}$	N	$\overline{T}-\overline{C}$	N	$\overline{T}-\overline{C}$	N	$\overline{T}-\overline{C}$	N
**Parents' beliefs on:**								
Impact of parents' inputs	0.82***	385	0.61***	284	0.61***	375	0.38***	323
	0.00^{pc}		*0.00*^{pc}		*0.00*^{pc}		*0.00*^{pc}	
	0.00^{ad}		*0.00*^{ad}		*0.00*^{ad}		*0.00*^{ad}	
Parental self-efficacy			-0.11	296	-0.03	377		
			0.56^{pc}		*0.73*^{pc}			
			0.56^{ad}		*0.73*^{ad}			
Intelligence malleability					0.10	367	-0.01	315
					0.37^{pc}		*0.94*^{pc}	
					0.59^{ad}		*0.94*^{ad}	
**Parents' inputs:**								

Continued on next page...

... table 4 continued

	6 months		7/9 months		12 months		18 months	
	$\overline{T}-\overline{C}$	N	$\overline{T}-\overline{C}$	N	$\overline{T}-\overline{C}$	N	$\overline{T}-\overline{C}$	N
Sensitivity to cues	-0.06	363	0.29**	341	0.19	374	-0.04	322
	0.60^{pc}		*0.01*^{pc}		*0.09*^{pc}		*0.73*^{pc}	
	0.60^{ad}		*0.05*^{ad}		*0.28*^{ad}		*0.97*^{ad}	
Response to distress	0.13	363	-0.06	341	0.16	374	0.05	322
	0.22^{pc}		*0.66*^{pc}		*0.25*^{pc}		*0.75*^{pc}	
	0.60^{ad}		*0.66*^{ad}		*0.44*^{ad}		*0.94*^{ad}	
SE growth fostering	0.09	363	0.19	341	0.12	374	0.02	322
	0.35^{pc}		*0.06*^{pc}		*0.19*^{pc}		*0.80*^{pc}	
	0.56^{ad}		*0.11*^{ad}		*0.46*^{ad}		*0.80*^{ad}	
Cognitive growth fostering	0.11	363	0.26*	341	-0.11	374	0.15	322
	0.30^{pc}		*0.04*^{pc}		*0.34*^{pc}		*0.17*^{pc}	
	0.63^{ad}		*0.10*^{ad}		*0.34*^{ad}		*0.48*^{ad}	
**Child outcomes:**								
Clarity of cues	-0.01	363	0.23*	341	-0.09	374	0.01	322
	0.92^{pc}		*0.03*^{pc}		*0.35*^{pc}		*0.93*^{pc}	
	0.92^{ad}		*0.06*^{ad}		*0.35*^{ad}		*0.93*^{ad}	
Responsiveness	-0.06	363	0.19*	341	-0.13	374	0.18	322
	0.53^{pc}		*0.07*^{pc}		*0.14*^{pc}		*0.09*^{pc}	
	0.76^{ad}		*0.07*^{ad}		*0.24*^{ad}		*0.16*^{ad}	
Words understood			0.19	319	0.22	369		
			0.15^{pc}		*0.15*^{pc}			
			0.26^{ad}		*0.25*^{ad}			
Words understood+said			-0.00	319	-0.00	369		
			1.00^{pc}		*0.98*^{pc}			
			1.00^{ad}		*0.98*^{ad}			
Words said							-0.10	321
							0.39^{pc}	
							0.60^{ad}	
Combines words							0.00	320
							0.94^{pc}	
							0.94^{ad}	

Note: $\overline{T}-\overline{C}$ gives the effect size of the intervention at 6, 7/9, 12 or 18 months. Below in italics are the per comparison (multiplicity-unadjusted) bootstrap p-value (pc), and the multiplicity-adjusted p-value (ad), both based on a two-sided Student's t test. The adjustment procedure follows List, Shaikh and Xu (2019). Families correspond to the set of four parental inputs, the set of two child's behaviors and the set of two vocabulary measures at each time point. N gives the number of observations used in the regression. In the 7/9 months column, beliefs are measured at 7 months, and behaviors are measured at 9 months. * for multiplicity-adjusted p-value<0.1, ** for multiplicity-adjusted p-value<0.05, *** for multiplicity-adjusted p-value<0.01.

Supplementary Table 5: Impacts of the home-visiting (HV) program at different time points

	30-36 months		36-42 months	
	$\overline{T}-\overline{C}$	N	$\overline{T}-\overline{C}$	N
**Parents' beliefs on:**				
Impact of parents' inputs	1.46***	69	1.25***	68
	0.00^{pc}		*0.00*^{pc}	
	0.00^{adj}		*0.00*^{adj}	

Continued on next page...

... table 5 continued

	30-36 months		36-42 months	
	$\overline{T}-\overline{C}$	N	$\overline{T}-\overline{C}$	N
Parental self-efficacy	0.25	59		
	0.17^{pc}			
	0.17^{adj}			
Intelligence malleability	1.05***	69		
	0.00^{pc}			
	0.00^{adj}			
**Child outcomes:**				
WM Verbal Analogies English			-0.01	61
			0.97^{pc}	
			0.97^{adj}	
WM Picture Vocabulary English			0.07	61
			0.77^{pc}	
			0.93^{adj}	
ROWPVT English			0.15	67
			0.52^{pc}	
			0.80^{adj}	
WM Verbal Analogies Spanish			0.44	61
			0.14^{pc}	
			0.38^{adj}	
WM Picture Vocabulary Spanish			0.67**	61
			0.01^{pc}	
			0.04^{adj}	
ROWPVT Spanish			0.49	67
			0.05^{pc}	
			0.20^{adj}	

Note: $\overline{T}-\overline{C}$ gives the effect size of the intervention. In italics are the per comparison (multiplicity-unadjusted) bootstrap p-value (pc), and the multiplicity-adjusted p-value (ad), both based on a two-sided Student's t test. The adjustment procedure follows List, Shaikh and Xu (2019). Families correspond to the set of belief measures used at each time point and to the set of six vocabulary measures for the 36-42-month assessment. N gives the number of observations used in the regression. * for multiplicity-adjusted p-value<0.1, ** for multiplicity-adjusted p-value<0.05, *** for multiplicity-adjusted p-value<0.01.

7 Robustness analysis

This section presents the average treatment effects (ATEs) discussed in the paper under two alternative specifications, with control variables and separating the two control subgroups for the Newborn experiment.

7.1 ATEs with controls

The choice of the control variables to add was dictated by two concerns: controlling for differences between the control and treatment groups that can occur due to small sample size and attrition, and increasing the precision of the estimates. To address both concerns, we rely on the method described in Belloni et al.[19]. The method consists of selecting the variables that best predict the treatment status with a LASSO regression (first concern), and then selecting the variables that best predict the outcome, with a LASSO regression as well (second concern). In the final regression (Supplementary Equation 1), we include the union of the two sets of selected variables as controls. The first LASSO regression was run at each assessment point to account for possible differences between the treatment and control groups that would arise with attrition.

Supplementary Table 6 below shows that impacts on parental beliefs, inputs, and child outcomes are very similar to those presented in the paper (Table 1) when we add control variables.

Supplementary Table 6: Impacts of the two experiments at different time points with controls

	Newborn program				HV program	
	6m	7/9m	12m	18m	30-36m	36-42m
**Parents' beliefs on:**						
Impact of parents' inputs	0.69***	0.53***	0.46***	0.36***	1.31***	1.12***
	0.00	*0.00*	*0.00*	*0.00*	*0.00*	*0.00*
	383	282	374	321	68	67
**Parents' inputs:**						
Interactions w/ child°	0.13	0.25**	0.12	0.08	0.86***	0.44
	0.24	*0.04*	*0.28*	*0.49*	*0.01*	*0.17*
	362	340	374	321	61	60
**Child's outcomes:**						
Interactions w/ parent	-0.05	0.21**	-0.11	0.12	0.66***	0.29
	0.59	*0.05*	*0.21*	*0.21*	*0.00*	*0.33*
	362	340	374	321	61	60
Vocabulary°		0.10	0.07	-0.02		0.35**
		0.31	*0.58*	*0.77*		*0.02*
		318	369	318		60
Math skills						0.48*
						0.05
						66
Social-emotional skills					0.49**	0.28**
					0.03	*0.04*
					69	67

Note: Differences between treatment and control group means are shown first for the two experiments at different time points. Below in italics are the corresponding p-values based on two-sided Student's t tests without adjustment for multiple comparisons. N gives the number of observations. In the 7/9 months column, beliefs are measured at 7 months, and behaviors are measured at 9 months. ° signals that the measurement tools used in the two experiments are different (see Section 4 of Supplementary Information). In the 7/9 months column, beliefs are measured at 7 months, and interactions are measured at 9 months. * for p-value<0.1, ** for p-value<0.05, *** for p-value<0.01.

7.2 ATEs distinguishing the two control groups (Newborn program)

In the Newborn program, the randomization was done in two stages. We first randomized parents between treatment and control groups. Then within the control group, half of the parents were randomized to watch a safety video (the topics covered were safety of the baby in the car, in case of a fall, fire, burn, choke, or poisoning), and the other half would not get any intervention and simply receive usual care at the clinic. The protocol followed for the safety videos was the same as the one we followed for the treatment videos. The rationale for having a placebo control group watching videos on a different topic was to test whether changes in parents' outcomes are simply driven by the attention they receive as part of the experiment or by the actual content of the video.

Table 1 in the paper merges the two control subgroups. Supplementary Table 7 below compares the treatment group to the control group who did not receive any video, and to the control group who received the safety video. Note that the p-value adjustment follows the same families as described in section 5, but the size of the families is multiplied by two here, as we have two control groups. The table shows that impacts on beliefs are similar when we distinguish the two control groups, confirming that changes in parental beliefs presented in the paper are driven by the content of the language videos. Results on parents' interactions with their child are similar when we separate the two control groups as well. However, the impacts on children's interactions tend to be slightly different when we distinguish between the two control groups. Supplementary Table 7 shows that children's behaviors are also positively affected in the safety control group at 9 months, so the treatment effects are higher when we compare the language treatment group to the usual care group only (0.4σ versus 0.2σ in Table 1).

Supplementary Table 7: Impacts of the Newborn program at different time points (separate control subgroups)

	6 months		7/9 months		12 months		18 months	
	$\overline{T}-\overline{C'}$	$\overline{S}-\overline{C'}$	$\overline{T}-\overline{C'}$	$\overline{S}-\overline{C'}$	$\overline{T}-\overline{C'}$	$\overline{S}-\overline{C'}$	$\overline{T}-\overline{C'}$	$\overline{S}-\overline{C'}$
**Parents' beliefs on:**								
Impact of parents' inputs	0.85***<br>*0.00*^{pc}<br>*0.00*^{ad}	0.05<br>*0.72*^{pc}<br>*0.72*^{ad}	0.54***<br>*0.00*^{pc}<br>*0.00*^{ad}	-0.13<br>*0.37*^{pc}<br>*0.69*^{ad}	0.63***<br>*0.00*^{pc}<br>*0.00*^{ad}	0.03<br>*0.81*^{pc}<br>*0.96*^{ad}	0.37*<br>*0.02*^{pc}<br>*0.05*^{ad}	-0.02<br>*0.90*^{pc}<br>*0.90*^{ad}
Parental self-efficacy			-0.05<br>*0.85*^{pc}<br>*0.85*^{ad}	0.12<br>*0.66*^{pc}<br>*0.84*^{ad}	0.06<br>*0.62*^{pc}<br>*0.94*^{ad}	0.18<br>*0.13*^{pc}<br>*0.44*^{ad}		
Intelligence malleability					0.02<br>*0.87*^{pc}<br>*0.87*^{ad}	-0.15<br>*0.31*^{pc}<br>*0.74*^{ad}	-0.18<br>*0.17*^{pc}<br>*0.31*^{ad}	-0.33*<br>*0.04*^{pc}<br>*0.10*^{ad}
**Parents' inputs:**								
Interactions w/ child	0.21<br>*0.11*^{pc}<br>*0.26*^{ad}	0.16<br>*0.28*^{pc}<br>*0.47*^{ad}	0.26<br>*0.09*^{pc}<br>*0.14*^{ad}	-0.02<br>*0.93*^{pc}<br>*0.93*^{ad}	0.13<br>*0.32*^{pc}<br>*0.74*^{ad}	0.06<br>*0.71*^{pc}<br>*0.71*^{ad}	0.05<br>*0.76*^{pc}<br>*0.76*^{ad}	-0.10<br>*0.55*^{pc}<br>*0.76*^{ad}
**Child's outcomes:**								

Continued on next page...

... table 7 continued

	6 months		7/9 months		12 months		18 months	
	$\overline{T}-\overline{C'}$	$\overline{S}-\overline{C'}$	$\overline{T}-\overline{C'}$	$\overline{S}-\overline{C'}$	$\overline{T}-\overline{C'}$	$\overline{S}-\overline{C'}$	$\overline{T}-\overline{C'}$	$\overline{S}-\overline{C'}$
Interactions	0.11	0.31	0.40**	0.33*	-0.09	0.09	0.18	0.10
w/ parent	0.39^{pc}	0.03^{pc}	0.01^{pc}	0.03^{pc}	0.43^{pc}	0.48^{pc}	0.16^{pc}	0.49^{pc}
	0.39^{ad}	0.11^{ad}	0.02^{ad}	0.09^{ad}	0.78^{ad}	0.73^{ad}	0.46^{ad}	0.85^{ad}
Vocabulary			0.15	0.11	0.21	0.19	-0.03	0.02
			0.16^{pc}	0.41^{pc}	0.15^{pc}	0.35^{pc}	0.73^{pc}	0.81^{pc}
			0.28^{ad}	0.41^{ad}	0.26^{ad}	0.35^{ad}	0.92^{ad}	0.81^{ad}

Note: $\overline{T}-\overline{C'}$ (resp. $\overline{S}-\overline{C'}$) gives the effect size of the intervention at 6, 7/9, 12 or 18 months for TMW (resp. Safety) video vs Usual care group. Below in italics are the per comparison (multiplicity-unadjusted) bootstrap p-value (pc), and the multiplicity-adjusted p-value (ad), both based on a two-sided Student's t test. The adjustment procedure follows List, Shaikh and Xu (2019). Families of outcomes for the adjustment are described in Section 5 of Supplementary Information. In the 7/9 months column, beliefs are measured at 7 months, and behaviors are measured at 9 months. * for multiplicity-adjusted p-value<0.1, ** for multiplicity-adjusted p-value<0.05, *** for multiplicity-adjusted p-value<0.01. The number of observations is the same as in Table 1 (for Parental self-efficacy and Intelligence malleability, please refer to Table A.4).

8 Experimenter demand effect

In our studies, experimenter demand effects could happen in two ways. First, the intervention that treated parents receive gives them cues about what the experimenter considers to be desirable attitudes, and the higher scores we observe in self-reported surveys could merely be due to parents learning about what we hope to find. While this could be true at the six-month assessment for the Newborn program, for example, which takes place right after parents watch the last video, it is less likely to persist in the following assessments. Yet, one year after the end of the intervention, at the eighteen-month assessments, treated parents still score 0.4σ higher than control parents. It should also be noted that in both experiments, it was made clear that answers to surveys would remain anonymous and be only analyzed in aggregation, which likely mitigated incentives for parents to try to please the experimenter.

Another way in which experimenter demand effects could happen is through the surveys. Asking parents about their attitudes with their child could indeed induce them to search for information about the topics covered in the survey and, if that searching behavior is asymmetric between treated and control parents (if control parents search more, for instance, because they do not receive information about child language development while the survey elicits their beliefs about it), our treatment effects would be biased (downward if control parents exhibit compensating behaviors). Nonetheless, Supplementary Figure 1 below shows that the evolution of beliefs in the control group between the baseline and following assessments is rather stable across both field experiments, suggesting that control parents do not update their beliefs over the period of study. Furthermore, this fear is attenuated when we consider the link between beliefs and actions.

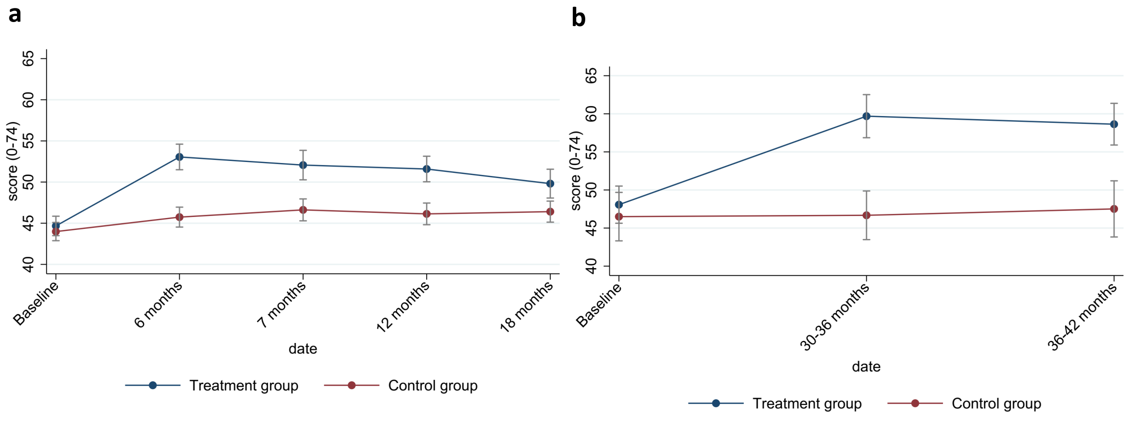

Supplementary Figure 1: **Evolution of parents' beliefs about how parental investments affect child development at different time points**. **a** Newborn program. **b** Home-visiting (HV) program. Mean scores with confidence intervals (CIs) in gray for each group. The mean scores and CIs for the treatment and control groups are provided as a Source Data file.

Supplementary References

1 Cunha, F. & Heckman, J. The technology of skill formation. *American Economic Review* **97**, 31–47 (2007).

2 Boneva, T. & Rauh, C. Human capital production and parental beliefs. *Unpublished manuscript* (2017).

3 Rowe, M. L. Child-directed speech: Relation to socioeconomic status, knowledge of child development and child vocabulary skill. *Journal of Child Language* **35**, 185–205 (2008).

4 Suskind, D. L. *et al.* Development of the Survey of Parent/Provider Expectations and Knowledge (SPEAK). *First Language* **38**, 312–331 (2018).

5 Cunha, F., Elo, I. & Culhane, J. Eliciting maternal expectations about the technology of cognitive skill formation. Tech. Rep., National Bureau of Economic Research (2013).

6 Attanasio, O., Cunha, F. & Jervis, P. Subjective parental beliefs. their measurement and role. Tech. Rep., National Bureau of Economic Research (2019).

7 Kendall, S. & Bloomfield, L. Developing and validating a tool to measure parenting self-efficacy. *Journal of Advanced Nursing* **51**, 174–181 (2005).

8 Bloomfield, L. & Kendall, S. Audit as evidence: the effectiveness of '123 magic' programmes. *Community Practitioner* (2010).

9 Dweck, C. S. *Self-theories: Their role in motivation, personality, and development* (Psychology Press, 2013).

10 Sumner, G. & Spietz, A. NCAST caregiver/parent-child interaction teaching manual. Seattle. *NCAST Publications* (1994).

11 Kolobe, T. H. Childrearing practices and developmental expectations for mexican-american mothers and the developmental status of their infants. *Physical Therapy* **84**, 439–453 (2004).

12 Lugo-Gil, J. & Tamis-LeMonda, C. S. Family resources and parenting quality: Links to children's cognitive development across the first 3 years. *Child Development* **79**, 1065–1085 (2008).

13 Fenson, L. *et al.* Short-form versions of the macarthur communicative development inventories. *Applied Psycholinguistics* **21**, 95–116 (2000).

14 Squires, J., Bricker, D. & Twombly, E. *The ASQ: SE user's guide: For the Ages & Stages Questionnaires: Social-emotional.* (Paul H Brookes Publishing, 2002).

15 Zimmerman, I. L., Steiner, V. G. & Pond. *PLS-5: Preschool Language Scales* (Pearson, 2013).

16 Woodcock, R. W., Muñoz-Sandoval, A. F., Ruef, M. L. & Alvarado, C. G. *Woodcock-Muñoz Language Survey–Revised* (Itasca, IL: Riverside, 2005).

17 Brownell, R. *Receptive and expressive one-word picture vocabulary tests* (Bloomington, MN: Pearson Assessments, 2010).

18 Dunn, L. M. & Dunn, D. M. *Peabody Picture Vocabulary Test, Fourth Edition* (Pearson Education, 2007).

19 Belloni, A., Chernozhukov, V. & Hansen, C. Inference on treatment effects after selection among high-dimensional controls. *The Review of Economic Studies* **81**, 608–650 (2014).

20 List, J. A., Shaikh, A. M. & Xu, Y. Multiple hypothesis testing in experimental economics. *Experimental Economics* **22**, 773–793 (2019).
