# Supplementary material for: Shifting parental beliefs about child development to foster parental investments and improve school readiness outcomes
Source: Nat Commun. 2021 Oct 1;12:5765. doi: 10.1038/s41467-021-25964-y (PMC8486787; doi:10.1038/s41467-021-25964-y)
Supplement: Supplementary file 3 — Reporting Summary [file 41467_2021_25964_MOESM3_ESM.pdf]

nature research

Corresponding author(s): Dana L. Suskind

Last updated by author(s): Jul 7, 2021

Reporting Summary

Nature Research wishes to improve the reproducibility of the work that we publish. This form provides structure for consistency and transparency in reporting. For further information on Nature Research policies, see our Editorial Policies and the Editorial Policy Checklist.

Statistics

For all statistical analyses, confirm that the following items are present in the figure legend, table legend, main text, or Methods section.

n/a	Confirmed	
☐	☒	The exact sample size (*n*) for each experimental group/condition, given as a discrete number and unit of measurement
☐	☒	A statement on whether measurements were taken from distinct samples or whether the same sample was measured repeatedly
☐	☒	The statistical test(s) used AND whether they are one- or two-sided<br>*Only common tests should be described solely by name; describe more complex techniques in the Methods section.*
☐	☒	A description of all covariates tested
☐	☒	A description of any assumptions or corrections, such as tests of normality and adjustment for multiple comparisons
☐	☒	A full description of the statistical parameters including central tendency (e.g. means) or other basic estimates (e.g. regression coefficient) AND variation (e.g. standard deviation) or associated estimates of uncertainty (e.g. confidence intervals)
☐	☒	For null hypothesis testing, the test statistic (e.g. F, t, r) with confidence intervals, effect sizes, degrees of freedom and P value noted<br>*Give P values as exact values whenever suitable.*
☒	☐	For Bayesian analysis, information on the choice of priors and Markov chain Monte Carlo settings
☒	☐	For hierarchical and complex designs, identification of the appropriate level for tests and full reporting of outcomes
☐	☒	Estimates of effect sizes (e.g. Cohen's *d*, Pearson's *r*), indicating how they were calculated

Our web collection on statistics for biologists contains articles on many of the points above.

Software and code

Policy information about availability of computer code

Data collection	REDCap was used to collect the data for the Newborn and HV studies, and the CRI platform was used for the Longitudinal study (http://cri.uchicago.edu/cri-developing-new-community-platform-with-thirty-million-words/).
Data analysis	The website Research Randomizer was used for the randomization for all studies. Stata 15 was used to analyze the data for all studies. No custom code has been developped for the data analysis.

For manuscripts utilizing custom algorithms or software that are central to the research but not yet described in published literature, software must be made available to editors and reviewers. We strongly encourage code deposition in a community repository (e.g. GitHub). See the Nature Research guidelines for submitting code & software for further information.

Data

Policy information about availability of data

All manuscripts must include a data availability statement. This statement should provide the following information, where applicable:
- Accession codes, unique identifiers, or web links for publicly available datasets
- A list of figures that have associated raw data
- A description of any restrictions on data availability

The datasets generated and/or analyzed during the current study are not publicly available due to the presence of Protected Health Information but all de-identified datasets will be made available upon request from the corresponding author to replicate the results. Source data are provided with this paper.

Field-specific reporting

Please select the one below that is the best fit for your research. If you are not sure, read the appropriate sections before making your selection.

☐ Life sciences ☒ Behavioural & social sciences ☐ Ecological, evolutionary & environmental sciences

For a reference copy of the document with all sections, see nature.com/documents/nr-reporting-summary-flat.pdf

Behavioural & social sciences study design

All studies must disclose on these points even when the disclosure is negative.

Study description	Quantitative experimental study using longitudinal data on parental beliefs, behaviors, and child outcomes
Research sample	Newborn study: Spanish or English-speaking low-SES parents of newborns recruited in 10 pediatric clinics of the Chicagoland area serving medically underserved, underinsured, or uninsured populations.<br>HV study: Spanish-speaking low-SES parents of 24 to 30 months old children recruited through postings in medical clinics, grocery stores, daycare facilities, community resource fairs, and public transportation in the Chicagoland area.<br>Longitudinal study: English-speaking low-SES parents of 13 to 16 months old children recruited through postings at child care centers, libraries, health clinics, local stores, public transportation, and community organizations serving low-income populations in the Chicagoland area.<br>Detailed recruitment procedures are provided in the Methods section.<br><br>We focus on low-SES families as there is ample evidence that parental investments and child outcomes are lower among those families, as explained in the paper. A series of demographic characteristics are provided in the Supplementary Information, section 3 to describe the population our samples are reprensentative of in more details.
Sampling strategy	Power calculations were made to determine the sample size. Those calculations are presented in the Methods section.<br>The sampling strategy was designed to enroll low-SES English-speaking and/or Spanish-speaking families living in the Chicagoland area. It was based on voluntary response sampling as explained above.
Data collection	Surveys filled out by parents were used to collect beliefs data (pen and paper or tablets were used at the clinic in the presence of a trained RA or an email was sent to complete it at home).<br>Video and audio recordings using the NCAST and LENA instruments, respectively, were used at the clinic in the presence of a trained RA (NCAST) and at home (LENA) to collect parental inputs.<br>Surveys filled out by parents (pen and paper or tablets at the clinic in the presence of a trained RA) and in-person assessments were used at home in the presence of a trained assessor to collect child outcomes (see Supplementary Table 3 for a complete description).<br>Assessors were not blind to experimental condition and study hypothesis.
Timing	For the newborn study, the first enrollment took place on June 20, 2016 and the last enrollment on July 31, 2017. For the home-visiting study, the first enrollment took place on April 17, 2017 and the last enrollment on July 9, 2018.<br>For each study, only one cohort was enrolled and we collected data at regular time points for that cohort (see the specific timelines in the Methods section), following the same time intervals for all participants (e.g., a participant enrolled in June 2016 in the newborn study would be surveyed again in December 2016 for the 6-month assessment while a participant enrolled in January 2017 would be surveyed again in July 2017).
Data exclusions	No data were excluded from the analyses
Non-participation	Non-participation rates are described in detail in the Methods section.
Randomization	Participants were randomly allocated to groups using Research Randomizer website. Additional details are provided in the Methods section.

Reporting for specific materials, systems and methods

We require information from authors about some types of materials, experimental systems and methods used in many studies. Here, indicate whether each material, system or method listed is relevant to your study. If you are not sure if a list item applies to your research, read the appropriate section before selecting a response.

Materials & experimental systems

n/a	Involved in the study
☒	☐ Antibodies
☒	☐ Eukaryotic cell lines
☒	☐ Palaeontology and archaeology
☒	☐ Animals and other organisms
☐	☒ Human research participants
☐	☒ Clinical data
☒	☐ Dual use research of concern

Methods

n/a	Involved in the study
☒	☐ ChIP-seq
☒	☐ Flow cytometry
☒	☐ MRI-based neuroimaging

Human research participants

Policy information about studies involving human research participants

Population characteristics	See above
Recruitment	Newborn study: parents were recruited in pediatric clinics by trained RAs.<br>HV study: parents were recruited through postings in medical clinics, grocery stores, daycare facilities, community resource fairs, and public transportation.<br>Longitudinal study: parents were recruited through postings at child care centers, libraries, health clinics, local stores, public transportation, and community organizations.<br><br>In the three cases, parents had to first volunteer to participate in the study, and then they were screened to check that they were eligible following the criteria described in the Methods section. The first step implies that only parents interested in the topic of the study were ultimately recruited. We do not believe that this self-selection creates any major issue for the interpretation of our results. The second step implies that our sample is representative of a specific population who presents the characteristics we used to determine eligibility. This is not an issue for the interpretation of our results as long as we do not extrapolate to other populations.
Ethics oversight	For all the studies involved in the analysis, we received approval from the University of Chicago Biological Sciences Division Institutional Review Board.

Note that full information on the approval of the study protocol must also be provided in the manuscript.

Clinical data

Policy information about clinical studies

All manuscripts should comply with the ICMJEguidelines for publication of clinical research and a completedCONSORT checklist must be included with all submissions.

Clinical trial registration	Newborn study: NCT02812017<br>HV study: NCT03076268<br>Longitudinal study: NCT02216032
Study protocol	The full study protocols can be accessed on clinicaltrials.gov:<br><br>Newborn study: https://clinicaltrials.gov/ct2/show/NCT02812017<br>HV study: https://clinicaltrials.gov/ct2/show/NCT03076268<br>Longitudinal study: https://clinicaltrials.gov/ct2/show/NCT02216032
Data collection	See above
Outcomes	Following our theory of change (described in the paper and in the Supplementary Information, section 1), our primary outcomes are parental beliefs, parental investments and child outcomes. We assess those measures comparing treatment and control groups via simple linear regressions.
